# Supplementary material for: Dominant forest tree species are potentially vulnerable to climate change over large portions of their range even at high latitudes
Source: PeerJ. 2016 Jul 13;4:e2218. doi: 10.7717/peerj.2218 (PMC4950616; doi:10.7717/peerj.2218)
Supplement: Table S1 — Climate anomalies were calculated for the modelling area. [file peerj-04-2218-s011.docx]

| Country | Center | Type | Model | SRES^[[1]](#footnote-1)^ | Member | Climate anomalies  (difference of a future climate to the 1961-1990 climate) | | | | | |
| --- | --- | --- | --- | --- | --- | --- | --- | --- | --- | --- | --- |
|  |  |  |  |  |  | 2050 | | | 2080 | | |
|  |  |  |  |  |  | TEM | PRE | PRATIO | TEM | PRE | PRATIO |
|  |  |  |  |  |  | °C | mm (%) |  | °C | mm (%) |  |
| Canada | Ouranos | regional | crcm 4.2.0 | a2 | cccma_cgcm3_1 run 4 | 2.8 | 66 (6) | -0.14 | 4.7 | 101 (10) | -0.25 |
| Australia | CSIRO Atmospheric Research | global | mk3.0 | a1b | run 1 | 1.7 | 37 (4) | -0.05 | 2.9 | 44 (4) | -0.09 |
| Germany / Korea | Meteorological Institute of the University of Bonn, Meteorological Research Institute of KMA, and Model and Data group. | global | miub_echo_g | b1 | run 1 | 2.3 | 25 (2) | 0.01 | 3.5 | 35 (3) | 0.00 |
|  |  |  |  | a2 | run 4 | 3.3 | 43 (4) | 0.03 | 5.1 | 91 (9) | 0.00 |
| Germany | Max Planck Institute for Meteorology | global | mpi_echam5 | a2 | run 1 | 2.2 | 115 (11) | -0.07 | 4.5 | 186 (18) | -0.09 |
| Japan | Center for Climate System Research, National Institute for Environmental Studies, and Frontier Research Center for Global Change (JAMSTEC) | global | miroc3_2_medres | a2 | run 1 | 3.5 | -22 (-2) | -0.15 | 6.6 | -90 (-9) | -0.31 |
| France | Institut Pierre Simon Laplace | global | ipsl_cm4 | a1b | run 1 | 4.1 | 20 (2) | -0.04 | 5.5 | 7 (1) | -0.07 |

1. SRES Special Report on Emissions Scenarios [↑](#footnote-ref-1)
