# Supplementary material for: Dominant forest tree species are potentially vulnerable to climate change over large portions of their range even at high latitudes
Source: PeerJ. 2016 Jul 13;4:e2218. doi: 10.7717/peerj.2218 (PMC4950616; doi:10.7717/peerj.2218)
Supplement: Table S2 — SRES used in this study are in bold. [file peerj-04-2218-s012.docx]

| RCP | Median temperature increase (ºC) 2100 horizon above pre-industrial values ^1^ | Comparison to SRES |
| --- | --- | --- |
| 2.6 | 1.5 | Lower than all SRES scenarios; closer to equilibrium in 2100 than the other scenarios. |
| 4.5 | 2.4 | Similar to **SRES B1** (2.5ºC)*. Median temperatures in RCP4.5 rise faster than in **SRES B1** until mid-century, and slower afterwards. |
| 6.0 | 3.0 | Similar to SRES B2 (3.0ºC)*. Lower than **SRES A1B** (3.5ºC)*. Median temperatures in RCP6 rise faster than in SRES B2 during the three decades between 2060 and 2090, and slower during other periods of the twenty-first century. |
| 8.5 | 4.9 | Similar to SRES A1FI (5.0ºC)*. Higher than **SRES A2** (4.2ºC)*. Median temperatures in RCP8.5 rise slower than in SRES A1FI during the period between 2035 and 2080, and faster during other periods of the twenty-first century. |

^1^ Source: Rogelj et al. 2013

^*^ Median temperature increase (ºC) 2100 horizon above pre-industrial values (Rogelj et al. 2013).

Reference

Rogelj J, Meinshausen M, and Knutti R. 2012. Global warming under old and new scenarios using IPCC climate sensitivity range estimates. *Nature Climate Change* 2:248-253. DOI: 10.1038/NCLIMATE1385
