# Supplementary material for: Dominant forest tree species are potentially vulnerable to climate change over large portions of their range even at high latitudes
Source: PeerJ. 2016 Jul 13;4:e2218. doi: 10.7717/peerj.2218 (PMC4950616; doi:10.7717/peerj.2218)
Supplement: Table S3 [file peerj-04-2218-s013.docx]

| Species | AUC | Sensitivity (%) | Specificity (%) |
| --- | --- | --- | --- |
| Black spruce | 0.980 | 93.6 | 93.6 |
| Balsam fir | 0.984 | 95.1 | 95.1 |
| White birch | 0.970 | 92.9 | 92.9 |
| Yellow birch | 0.939 | 88.8 | 88.8 |
| Sugar maple | 0.916 | 86.6 | 86.5 |
